# Exogenous Isoprene Confers Physiological Benefits in a Negligible Isoprene Emitter (*Acer monspessulanum* L.) under Water Deficit

**DOI:** 10.3390/plants9020159

**Published:** 2020-01-28

**Authors:** Elena Ormeño, Justine Viros, Jean-Philippe Mévy, Alain Tonetto, Amélie Saunier, Anne Bousquet-Mélou, Catherine Fernandez

**Affiliations:** 1 CNRS, Aix Marseille Univ, Avignon Univ, IRD, IMBE, 13331 Marseille, France; justine.viros@imbe.fr (J.V.); jean-philippe.mevy@imbe.fr (J.-P.M.); anne.bousquet-melou@imbe.fr (A.B.-M.); catherine.fernandez@imbe.fr (C.F.); 2 Platform of analytical and technological research and imaging, FR1739, CNRS, Aix-Marseille Univ, Centrale Marseille, 13003 Marseille, France; alain.tonetto@univ-amu.fr; 3 Department of Environmental and Biological Sciences, University of Eastern Finland, P.O. Box 1627, 70211 Kuopio, Finland; amelie_saunier@outlook.fr

**Keywords:** isoprene protection, water deficit, oxidative pressure, negligible terpene emitters, abiotic stress, reactive oxygen species, allelochemicals

## Abstract

Isoprene, the main volatile released by plants, is known to protect the photosynthetic apparatus in isoprene emitters submitted to oxidative pressures caused by environmental constraints. Whether ambient isoprene contributes to protect negligible plant emitters under abiotic stress conditions is less clear, and no study has tested if ambient isoprene is beneficial during drought periods in plant species that naturally release negligible isoprene emissions. This study examines the effect of exogenous isoprene (20 ppbv) on net photosynthesis, stomatal conductance and production of H_2_O_2_ (a reactive oxygen species: ROS) in leaves of *Acer monspessulanum* (a negligible isoprene emitter) submitted to three watering treatments (optimal, moderate water stress and severe water stress). Results showed that *A. monspessulanum* exhibited a net photosynthesis increase (+30%) and a relative leaf H_2_O_2_ decrease when saplings were exposed to an enriched isoprene atmosphere compared to isoprene-free conditions under moderate water deficit. Such physiological improvement under isoprene exposure was not observed under optimal watering or severe water stress. These findings suggest that when negligible isoprene emitters are surrounded by a very high concentration of isoprene in the ambient air, some plant protection mechanism occurs under moderate water deficit probably related to protection against ROS damage eventually impeding photosynthesis drop.

## 1. Introduction

All plant species emit volatile organic metabolites, so called biogenic volatile organic compounds (BVOCs), with different emission capacities and different emission types according to plant species [1,2]. Terpene volatiles (also referred to as isoprenoids) form the largest class of BVOCs, although isoprene (C_5_H_8_, or 2-methyl-1,3-butadiene)—emitted in high amounts by numerous plant species such as *Quercus pubescens* Wild., *Quercus robur* L. or *Populus* spp., [1,2]—represents 70% of global plant emissions (530 Tg.y^−1^), followed by monoterpenes (C_10_H_16_) with 84 Tg.y^−1^ [3]. Isoprene is suggested to serve multiple purposes in plants, including protection of the leaf photosynthetic machinery from abiotic oxidant stressors (light, temperature, air pollution) [4], promotion of flowering in neighboring plants and interactions between plants and herbivores [5].

The role of isoprene in protecting both the emitting plant and neighboring plants during abiotic stresses is, however, under debate, as recently reviewed [6]. In isoprene-emitting plants, the basis of this debate (and even skepticism) relies on the fact that isoprene, as other BVOCs, is unavoidably released through the leaf own to its physicochemical properties. Indeed, once isoprene is synthesized within leaves, emissions occur due to its high vapor pressure and high isoprene cellular concentrations compared to trace concentrations of isoprene in ambient. Isoprene emission, hence, does not necessarily involve a functional role for the emitter plant [7].

The functional role of isoprene for isoprene-emitting plants and negligible isoprene emitters (often considered as non-isoprene-emitting plants) has been clarified over these two last decades, through the development of both (i) genetically modified plants to produce or suppress isoprene and (ii) isoprene chemical inhibitors. First, transgenic lines of a given species genetically modified to be able to emit isoprene have been compared with the natural species free of BVOC emissions [8]. This approach has showed that isoprene contributes to stabilize the thylakoid membrane in the transgenic lines of *Arabidopsis* sp. during severe heating [8]. Under water deficit conditions, transgenic lines of tobacco (*Nicotiana tabacum* L.) with the highest isoprene emission rates showed lower reactive oxygen species (ROS) content (in terms of H_2_O_2_) compared to the transgenic line featuring the lowest isoprene emissions [9]. Second, cut leaves treated with chemical isoprene inhibitors such as fosmidomicyn showed triggered ROS formation and poor photosynthesis under photoinhibition and severe heating, compared to leaves that naturally emit isoprene or that receive an exogenous isoprene supply [8,10,11,12]. In oak species that naturally produce isoprene, blocking isoprene biosynthesis through chemical inhibitors also limits heat tolerance compared to isoprene-fed leaves [12]. Likewise, isoprene-fed leaves are less sensitive to cell oxidation upon exposure to high ozone concentrations than isoprene-free leaves after chemical isoprene inhibition [12,13,14]. All these studies have provided strong evidence about the benefits of isoprene emissions in transgenic or natural isoprene emitters under high light, temperature, water deficit and ozone concentrations.

Studies about the effect of isoprene exposure in plant species that are not able to emit isoprene (or in very few amounts) are especially scarce. For example, it has been proven that heat tolerance increased in leaves of *Phaseolus vulgaris* L. and *Quercus ilex* L. when they were supplied with exogenous isoprene while exposed to very high temperatures (35–46 °C) for a short period (in the order of minutes) [15,16]. Testing whether an atmosphere rich in isoprene protects natural species that release little or negligible isoprene emissions (such as *Acer* spp., [17]) during water deficit is of special importance in the Mediterranean region where species naturally cope with drought periods in summer, and climate predictions point towards an increasing aridity by the end of this century [18]. Physiological benefits conferred by isoprene in non-isoprene emitters could occur under natural conditions as far as they grow intermixed with isoprene emitters. Leaves from non-isoprene emitters can potentially take up isoprene (or other volatiles) from the ambient air because of the isoprene concentration gradient between the surrounding air (with high isoprene concentration) and the leaf tissues (with negligible isoprene concentrations) promoting isoprene concentration within leaves of non-terpene emitters [19,20]. Rapid metabolization of isoprene (within cellular destruction) taken up by leaves from non-isoprene emitters maintains the isoprene concentration gradient and explains the sustainability of this process [21]. Exchanges of volatiles between plants and the atmosphere are thereby bidirectional (and not unidirectional as most often assumed, see citations from [21]). Emissions of isoprene from leaves to the atmosphere would dominate in isoprene emitters, while isoprene uptake (from the atmosphere to the leaf) would potentially dominate in non-terpene emitters [21].

In Mediterranean forests, two tree species, *Acer monspessulanum* L. and *Quercus pubescens* Wild., coexist together in mature forests with a remarkable tree density in some forests [17] implying that their canopies are intertwined. Both species are also known for their remarkable resistance to drought [22,23] while they feature contrasting capacities to emit isoprene. *A. monspessulanum* releases negligible isoprene emission rates (0.3 µg.g^−1^.h^−1^) or any other terpene [17], while *Q. pubescen*s is the major isoprene emitter in the Mediterranean region (~100 µg.g^-1^.h^−1^) [24]. This situation raises the question of the possible protection an isoprene-enriched atmosphere could confer to *A. monspessulanum* under water deficit in Mediterranean forests. Based on previous literature, we hypothesize that isoprene released by *Q. pubescens* in the atmosphere could protect the photosynthetic machinery of the non-emitter *A. monspessulanum* through an attenuation of the oxidative pressure. In order to test this hypothesis, we explore whether exogenous isoprene confers physiological protection to *A. monspessulanum* (assessed through ROS and photosynthesis) under water stress in laboratory conditions.

## 2. Results

### 2.1. Verification of the Negligible Terpene Emissions from A. monspessulanum

As expected, *A. monspessulanum* saplings released negligible isoprene emissions (m/z 69, 0.08 µg.g^-1^.h^-1^). Non-significant emission rates of non-oxygenated monoterpenes (m/z 137, 0.04 µg.g^-1^.h^-1^) and oxygenated monoterpenes (m/z 153, 0.01 µg.g^-1^.h^-1^) were also detected.

### 2.2. Variation of Physiological Traits According to Watering and Fumigation Treatments

**Figure 1:**
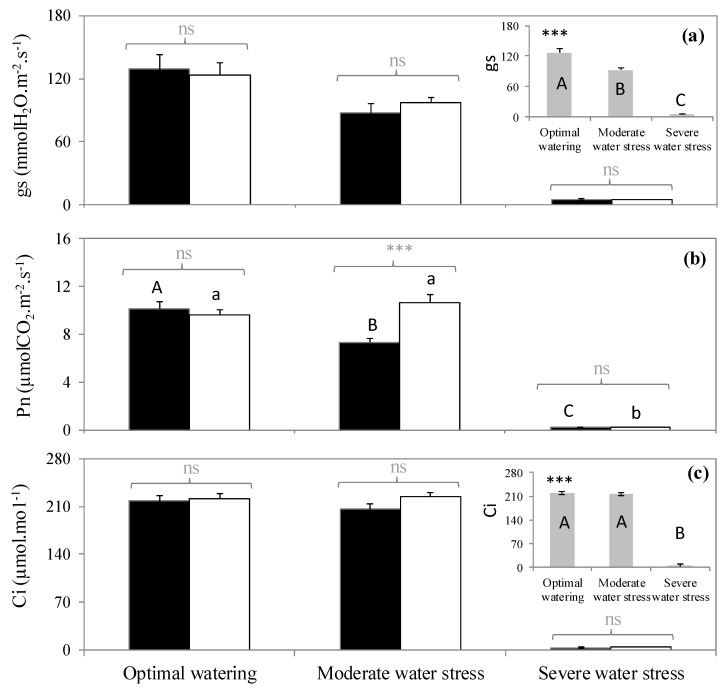
(**a**) Stomatal conductance to water, (**b**) net photosynthesis, and (**c**) intercellular CO_2_ concentration in three watering and two fumigation treatments. *A* > *B* > *C*: significant differences among watering treatments following Tukey tests when interactions between fumigation and watering were not significant (embedded graphs). Embedded graphs reflect the absence of significant interaction between watering and fumigation (changes of *gs* and *Ci* were the same independently of the fumigation condition). *A > B > C*: significant differences between watering treatments following Tukey test within the isoprene-free fumigation treatment. a > b: significant differences between water treatments following Tukey test within the isoprene fumigation treatment. Differences between fumigation treatments (noted in grey above brackets) within each watering treatment are tested with Student tests (***: *p* < 0.001, ns: not significant differences). Values are mean ± se, *n* = 5 (*n*: number of saplings).

The three watering treatments represented three clearly distinct plant water levels (*p* < 0.001, Table 1). Optimally watered saplings did not exhibit water deficit signs either in terms of stem water potential (ψ = −0.7 MPa) or leaf water content (56%). These parameters decreased with increasing water withholding. Thus, intermediate values were reached under moderate water stress (ψ = −2.7 MPa, leaf water content = 20.6%), and very low values were obtained in severely stressed saplings, which exhibited a dramatic drop of ψ (−5.7 MPa) and only 7% of leaf water content (Table 1). Moreover, stomatal conductance to water vapor (*gs*) decreased as water deficit increased (Figure 1a) independently of the fumigation treatment (no significant interaction between watering and fumigation, *p* > 0.05, Table 2) with a moderate reduction of *gs* under moderate water stress and stomatal closure in severely water stress saplings leading to insignificant *gs* values (Figure 1a).

Contrastingly, net photosynthesis (*Pn*) decreased as water stress increased, but only in the isoprene-free fumigation treatment (*p* < 0.001, Figure 1b), indicating a significant interaction between fumigation and water stress (Table 2). In the isoprene-free fumigation conditions, *Pn* decreased by 30% in leaves subjected to moderate water stress and was mostly inhibited under severe water stress following stomatal closure as indicated by the insignificant intercellular CO_2_ concentration (*Ci*). However, under exogenous isoprene, *Pn* remained similar in the moderate water stress plants and optimally watered saplings. As a result, *Pn* of moderate water stress plants was 30% higher when they had been treated with exogenous isoprene compared to isoprene-free fumigation (Figure 1a). This improvement occurred despite *gs* being similar when plants were or were not exposed to isoprene (*p* < 0.05, Figure 1a), indicating that *Pn* maintenance under moderate water stress when plants were exposed to isoprene was not associated or driven by *gs*. Instead, exposure to isoprene was associated to a concomitant reduction of ROS pressure (see below). By contrast, *Pn* improvement in plants exposed to isoprene did not occur under optimal watering and severe water stress (*p >* 0.05, Figure 1b).

### 2.3. Variation of the Oxidative Pressure (H_2_O_2_) According to Watering and Fumigation Treatments

**Figure 2:**
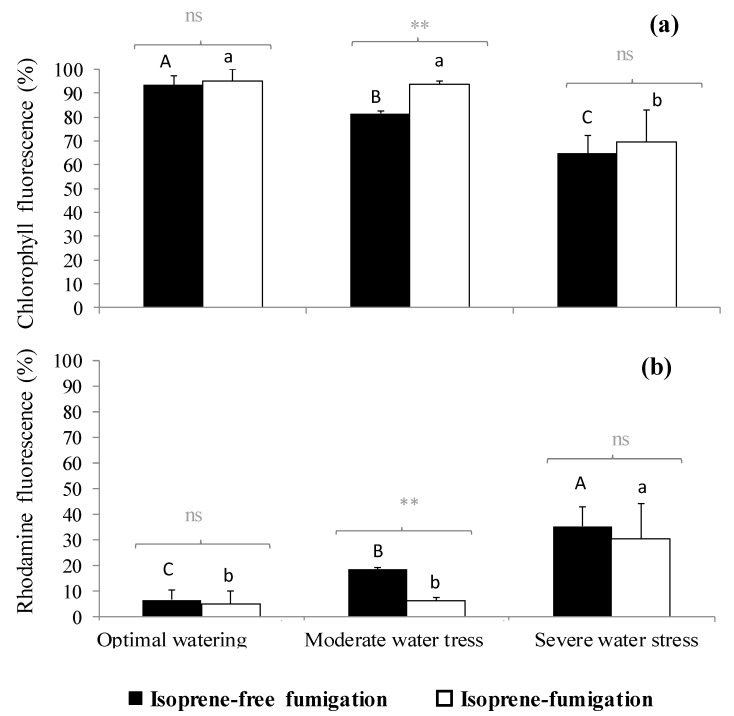
(**a**) Chlorophyll fluorescence intensity and (**b**) rhodamine autofluorescence (expressed in percentage with respect to total fluorescence, so the sum of both fluorescence intensities reaches 100%) under three watering and two fumigation treatments. Differences between fumigated treatments (noted in grey above brackets) within each watering treatment are tested through Mann–Whitney tests (**: 0.001 < *p* < 0.01, ns: not significant differences). Differences between watering treatments within each fumigation treatment are tested through Kruskal–Wallis tests and are denoted with different letters (*A* > *B* > *C* for isoprene-free fumigation, *a* > *b* for isoprene fumigation). Values are mean ± se, *n* = 3 (n: number of saplings).

**Figure 3:**
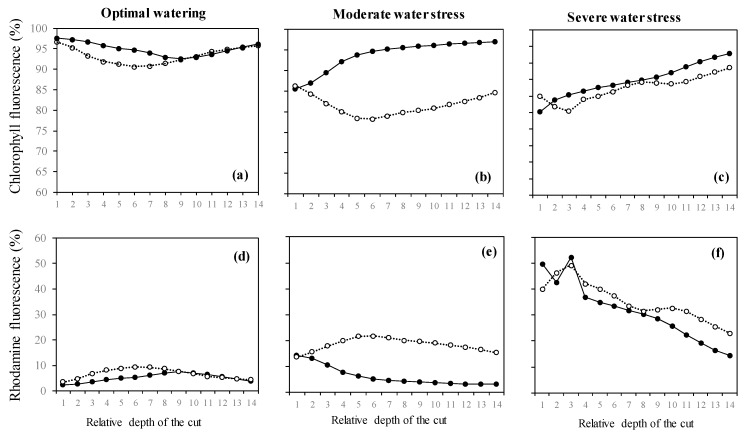
(**a–c**) Intensities of chlorophyll fluorescence and (**d–f**) intensities of rhodamine fluorescence (denoting H_2_O_2_ presence) from leaves of *Acer* *monspessulanum* across 14 leaf cuts carried out through laser penetration. Values are mean with *n* = 3 (*n*: number of saplings).

**Figure 4:**
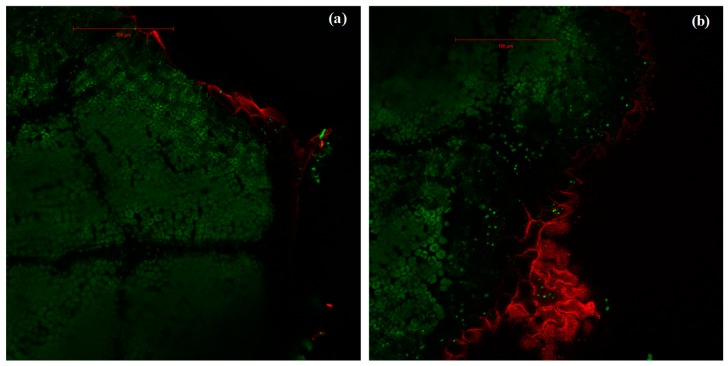
Intensities of rhodamine fluorescence (in red) and chlorophyll autofluorescence (green) in leaves of *A. monspessulanum.* (**a**) Leaf fumigated with isoprene where rhodamine fluorescence, marker of H_2_O_2_ pressure, occurs only in the epidermis; (**b**) leaf not fumigated with isoprene where rhodamine occurs both superficially (cuticle and epidermis) and in deeper mesophyll tissue. Images correspond to section 6 from a moderate stress sapling leaf.

In isoprene-free fumigated leaves, chlorophyll fluorescence decreased with water deficit (*p* < 0.001, Figure 2a, Figure 3a,b,c). Accordingly, the H_2_O_2-_related oxidative pressure, assessed through rhodamine fluorescence, increased proportionally to water deficit as shown by the low (5.0%), intermediate (18.5%) and high values of rhodamine fluorescence intensity (35.3%) under optimal watering, moderate and severe water stress, respectively (Figure 2b, *p* < 0.001, averaged fluorescence intensities, Figure 3d,e,f). Contrastingly, under exogenous isoprene and moderate water stress, plants exhibited the same relative amount of H_2_O_2_ than optimally watered saplings (Figure 2b). The relative amount of H_2_O_2_ only dramatically increased under severe water stress independently of the fumigation treatment. Likewise, analysis of fluorescence intensities through leaf cuts in moderately water-stressed saplings showed that rhodamine values were lower in the isoprene fumigation treatment independently of the leaf cut (from cut 2 to cut 14, Figure 3e) and thus from leaf cuticle and epidermis to deeper mesophyll cells (Figure 4). Accordingly, chlorophyll fluorescence was higher under isoprene supply in all leaf tissues (Figure 3b). This limited ROS pressure after isoprene fumigation was not observed either in the optimal watering or severe water stress, as indicated by the similar averaged fluorescence intensities of rhodamine in the two fumigation treatments within each of these watering treatments (Figure 2). Analysis of fluorescence layer by layer (Figure 3) allowed, however, to evidence that isoprene exposure was actually associated to a slightly lower rhodamine fluorescence (and so limited presence of H_2_O_2_) in many (but not all) leaf cuts, both in optimal watering (Figure 3d) and sever stress (Figure 3f).

## 3. Discussion

Results showed in this study suggest that exposure of *A. monspessulanum* leaves to an enriched isoprene atmosphere improves leaf functioning (net CO_2_ fixation) and limits oxidative pressure (H_2_O_2_) under moderate water stress. Such improvement is not driven by stomata opening, since isoprene fumigation did not influence *gs* or *Ci*. Instead, isoprene seems to protect the leaf photosynthetic apparatus by limiting the oxidative pressure (H_2_O_2_) in leaf cells, from the superficial leaf layers (cuticle and epidermis) to the deep leaf layers (mesophyll tissues). By contrast, isoprene fumigation did not modify photosynthetic rates in *A. monspessulanum* either in unstressed (optimal watering) or severe water stress conditions. In these two drought conditions, H_2_O_2_ formation was only limited in isoprene-fumigated plants in some foliage cuts and not all throughout the leaf depth.

From an ecological perspective, the remarkable air concentrations of isoprene applied to plants in this study (20 ppbv) have been detected in the ambient atmosphere of the canopy in a *Q. pubescens*-dominated forest and co-dominated by *A. monspessulanum*, during summer when drought stress was most remarkable [25] and even during punctual hot days in late spring at midday [26]. Given the intertwined canopies of these two species in the forest, and the high isoprene concentrations reached herein effectively protected foliage photosynthetic apparatus from moderate water depletion in this study, our data indicate that such benefit could occur under natural conditions upon drought and hot periods. Coexistence of *A. monspessulanum* with high isoprene emitters (such as *Q. pubescens*) may increase drought resistance of *A. monspessulanum.* This species grows in drier zones in France and shows a higher resistance to water deficit compared to other *Acer* species that occur in more humid areas (*A. pseudoplatanus*, *A. negundo*, *A. platanoides*, *A. campestre)* [22], [25]. Such resistance is reflected in cavitation of *A. monspessulanum,* which occurs in the field under very low ψ values (between −3 MPa and −4.5 MPa corresponding to beginning and full cavitation, respectively) while it occurs between −1.2 and −2.75 MPa in the other *Acer* spp. more vulnerable to drought [22]. Accordingly, since ψ measured in this study was −2.65 and −5.66 MPa in moderately and severely stressed saplings respectively, we can conclude that the applied watering treatments are equivalent to moderate and very severe water stress in the field.

Several findings reveal that exogenous isoprene and monoterpenes have a positive effect in both non-terpene emitters and terpene emitters (previously inhibited to synthesize terpenes), through photosynthesis improvement under extreme heating [15,27,28,29,30,31], acute ozone concentrations [13] and photoinhibition [11]. It has been argued that such benefits occur under both low and high atmospheric monoterpene concentrations [30,31,32], whereas only exposure to high isoprene concentrations would be beneficial [29,31]. The later study explains such difference by their lipid phase to gas phase partition coefficient assessed through the ratio between octanol/water partition coefficient (*Kow*) and Henry’s low constant (*H*). Such ratio is 75-fold higher for isoprene than monoterpenes, and, as a result, concentration of isoprene supplied to plants needs to be 75-fold larger than for monoterpenes to achieve the same concentration within leaf membranes. All these studies suggest that in the Mediterranean region, where numerous species present the capacity to release intermediate to high terpene emission rates [2], their isoprenoids could confer protection against oxidative stress in neighboring negligible emitters too, and that this process might be the rule rather than the exception.

How isoprene helps plants to face oxidative pressures is still under discussion [6], although three modes of action (hereafter synthesized) are frequently put forward: energy dissipation, stabilization of thylakoid membranes and ROS scavenging.

First, isoprene seems to assist plant species, especially those from temperature regions, by protecting their photosynthetic apparatus through energy dissipation during transient and moderate stresses, rather than extreme environmental stresses [31]. Under temperatures in the range of 30–35 °C (physiologically high temperatures), leaves show an enhancement of heat dissipation in chloroplasts of isoprene-emitting species after chemical or genetic manipulations (*Arabidopsis* spp., *Nicotiana* spp. and *Populus* spp.) [32]. Since restoration of endogenous isoprene concentration through exogenous isoprene supply did not restore the energy dissipation (non-photochemical quenching of chlorophyll fluorescence) to values as low as in isoprene-emitting leaves, these authors suggest that benefits conferred by isoprene due to isoprene-related energy dissipation only occur in terpene emitters. Cost of isoprene synthesis in plant emitters would thus be counterbalanced by the evolutionary advantage isoprene confers to them.

Second, it has been suggested that isoprene could result in the stabilization of thylakoid membranes in chloroplasts, thus impeding their denaturation under stress conditions, despite the high volatility and short residence time of isoprene in the thylakoid membrane [10,33]. This hypothesis relies on the lipophilic character of isoprene which can solubilize in lipid membranes. Accordingly, the use of simulation techniques in molecular dynamics has revealed that isoprene spontaneously partitions into the membrane bilayer where it accumulates at the center, resulting in a membrane similar to that observed under lowering temperatures [34].

Finally, another plausible explanation is that isoprene would be beneficial for the emitting plant through ROS scavenging, as far as the stress induces a significant ROS formation in leaves, indicating an isoprene antioxidant action, protecting *in fine* the photosynthetic machinery. The role of isoprene on scavenging the singlet delta oxygen (^1^ O_2_) is, so far, the best documented. This role is of special importance since ^1^ O_2_ is the ROS that most induces chloroplast degradation. Whilst this action is not necessarily restricted to isoprene, this volatile probably provides a more dynamic protection because of its prompt synthesis when high light (in the order of 1800 µmol. m^−2^. s^−1^) intensity produces ^1^ O_2_. Some authors go further into this hypothesis by suggesting that protection against ^1^ O_2_ formed during ultraviolet excitation would be the primary role of isoprene, rather than providing heat protection or scavenging against any other oxidant species (O_3_, OH^.^, H_2_O_2_) [35]. Vickers et al. [36] suggest, instead, a unified mechanism of action of all plant volatiles, where BVOCs would scavenge ROS conferring protection under any abiotic and biotic stress condition leading to oxidative pressure. Plant volatiles could hence counteract oxidative pressure during water deficit too, explaining why isoprene emissions represent a loss of up to 30%–50% of recently assimilated carbon from photosynthesis under water stress, while this percentage is only ~1% under optimal water supply [37].

In our study, we suggest that under moderate water stress, isoprene conferred protection against H_2_O_2_ resulting in improvement of *Pn*. Under these coupled conditions (isoprene fumigation under moderate water stress), we observed higher chlorophyll autofluorescence and poor intracellular H_2_O_2_ presence (low re-emitted rhodamine fluorescence) both in superficial leaf layers and internal leaf cells from the mesophyll. The underlying mechanism is probably related to ROS scavenging, as pointed out in past studies, although the approach applied in this study does not allow to isolate the different isoprene-related protective mechanisms (stabilization of thylakoid membranes, energy dissipation).

Benefits conferred by isoprene implicate that isoprene was taken up by leaves of *A. monspessulanum* (although isoprene concentration within leaves was not measured in the present study) to reduce the isoprene concentration difference between air surrounding the leaves and the foliage. As extensively researched in a recent study [21], such process is sustained by isoprene metabolization through oxidation reactions within leaves (so-defined as the isoprene chemical sink), rather than dissolution and storage of isoprene within the lipid phase of the leaf (so-defined as the physicochemical sink), since isoprene is hardly stored in the leaf lipophilic phase, as indicated by the relatively low octanol/water partition coefficient [28].

## 4. Materials and Methods

### 4.1. Acer Monspessulanum Saplings

We used *A. monspessulanum* saplings (2 years old) grown in our greenhouse facilities in pots (volume: 1.2 L) with natural soil from a forest site (Oak observatory at Observatory of Haute Provence, in Southern France). For soil and site description see Santonja et al. [38].

### 4.2. Watering Treatments

During two months (February 15th–April 20th) (during which isoprene fumigation was not performed), 42 samplings were submitted to 3 watering treatments (*n* = 14 for each treatment). At the beginning of the experiment (February) saplings were in dormancy, leaf burst occurred beginning March and the first developed leaves were visible beginning April. Optimally watered samplings were watered every two days to field water capacity (0.5 L). In moderately water-stressed samplings and severely water-stressed samplings, watering was applied following the same protocol, but watering was stopped at 7 and 13 days respectively.

Before fumigation was carried out, it was checked out that the three watering treatments implied three different water levels in terms of ψ and leaf water content. Measurements of ψ were carried out with a pressure chamber (Scholander, PMS instrument Co. Oregon USA) using two different stems from each sapling. A third stem was used when the ψ difference between the two first stems exceeded 0.2 MPa. Leaf water content (or leaf humidity) was calculated as 100 × ((fresh mass−dry mass)/(fresh mass)). A set of 4 saplings per treatment were used to measure leaf water content and ψ. Since these were destructive measurements, these 12 saplings (4 saplings × 3 watering treatments) were not used for any other purpose.

### 4.3. Acer Monspessulanum Fumigation Treatments

Fumigation and the subsequent physiological measurements took place from April 21st to May 15th. During this period, saplings featured approximately 30 developed leaves each and very similar height, with an average of 62.3 ± 3.2 cm (standard deviation).

Five saplings from each watering treatment were submitted to isoprene-fumigation and 5 others to isoprene-free fumigation, totalling 30 saplings used for fumigation treatments (5 saplings × 3 watering treatments × 2 fumigation treatments). This experimental design (the use of different saplings during isoprene-free and isoprene fumigation) was chosen since after each type of fumigation and once physiological measurements were accomplished, several leaves were cut to be immediately analyzed in terms of oxidative pressure (cf. 4.4). Such destructive action in isoprene-free fumigated saplings would have implied an additional mechanical stress in isoprene-fumigated plants.

**Figure 5:**
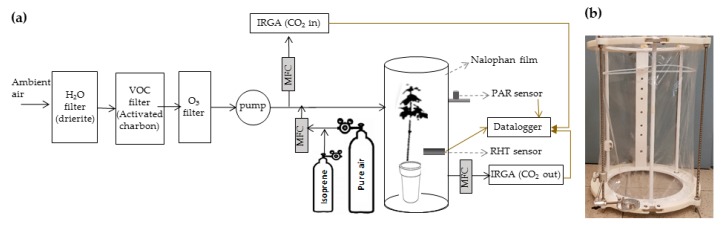
(**a**) Scheme of the system used during fumigation of a maple sapling. Continuous, discontinuous and brown arrows denote the flow direction, the name of the instrument and electronic connections respectively. MFC: Mass flow controller. (**b**) Picture of the enclosure chamber built with a PTFE-made structure and external stainless-steel bars (grey) for chamber fixation.

During fumigation, saplings were enclosed in two dynamics chambers of 77 L volume (77 cm height, 35 cm diameter) and PTFE-made (Polytetrafluoroethylene), based on our previous models [17,22,23] (Figure 5) (Figure 5a,b). Each chamber was enclosed using a Nalophan film (MediSense, UK). Ambient air was continuously flushed in each chamber using a mass flow controller (Bronkhorst, France) and a PTFE pump (KNF N840.1.1FT.18, Germany). Flushed ambient air contained a CO_2_ concentration ranging between 410 and 420 ppb, measured with an infrared gas analyzer (IRGA 840A, LI-COR, USA). This air was previously ozone filtered using a PTFE membrane filter conditioned with sodium thiosulfate (Na_2_S_2_O_3_) (Pollmann et al. 2005) to prevent BVOC oxidation inside the chamber and ozone-related oxidation in plants. Air was also BVOC filtered using activated charcoal (untreated, granular, 4–8, Honeywell Fluka). Light was supplied at the top of each chamber using metal-halogen lamps (OSRAM Powerstar HQI-BT 400W/D Pro Daylight E40, France). They provided an averaged photosynthetically active radiation (PAR) of 472.5 ± 26.8 µmol.m^−2^.s^−1^ during the day (LI-COR LI-190R Quantum Sensor, USA). Temperature and relative humidity inside each chamber were measured with RHT (relative humidity and temperature) sensors (HMP60, Vaisala, Finland). Daily temperature and relative humidity inside the dynamic chambers were 26.0 ± 0.4 °C and 74.8% ± 9.2% respectively (values are mean ± standard deviation). Temperature, relative humidity and PAR were recorded with a LI-COR 1400 datalogger.

In the isoprene-free fumigation treatment, each sapling was flushed with isoprene-free air for 24 h with a continuous flow of 1 L.min^−1^. In the isoprene-fumigation treatment, the same flow was supplied for 24 h with an additional supply of isoprene from a gaseous tank (3 ppm isoprene gas standard, Airliquide, France) previously diluted, resulting in an isoprene concentration in the inflow airstream of 20 ± 3 ppbv (mean ± standard deviation). This concentration, similar to that measured in the field [25,26] (cf. Discussion), was measured by directing 80 mL.min^−1^ of the continuous air flow to a commercial PTR-ToF-MS 8000 instrument (Ionicon Analytik GmbH, Innsbruck, Austria).

To check that fumigation was actually applied in negligible-isoprene emitters as assumed in literature, we monitored BVOC emissions at the branch level using the dynamic enclosure system previously described [39], connected to a PTR-ToF-MS 8000. Emissions were measured at 25 °C temperature and 700 µmol.s^−1^.h^−1^ PAR in a set of four saplings, previous to the study.

### 4.4. Physiological Performances of A. Monspessulanum at the Leaf Scale

Gas exchanges of CO_2_ and H_2_O were measured in 3 leaves for each sapling after sapling removal from the fumigation enclosures. Each leaf was clipped in a PLC3 Universal Cuvette (diameter 18 mm, 2.5 cm^2^ surface) relayed to an open-flow infrared gas analyzer equipped with a red-blue light source (Synersy, CIRAS 3 PPSystem, USA). Gas-exchange measurements (~5 min/leaf) allowed direct calculations of *Pn*, *gs* and *Ci* using the instrumental software. Measurements were performed under 30 °C temperature and constant CO_2_ concentration (400 ppm) using a CO_2_ mixer and saturating PAR (1000 µmol.m^−2^.s^−1^).

### 4.5. Estimation of ROS Presence Using Laser-Scanning Confocal Microscopy

After fumigation, ROS semi-quantification was analyzed in three saplings from the isoprene fumigation treatment and three others from the isoprene-free fumigation treatment and within each watering treatment. For each sapling, three leaves were used to assess ROS presence through hydrogen peroxide (H_2_O_2_). The average of these three values was then calculated for statistical purposes. Leaves were detached and a surface of 0.5 cm² was selected within each leaf. Then, a pinhole was made with a thin needle (diameter 0.5 µm) under a stereo microscope. The pinhole-made leaf samples were incubated for 30 min at room temperature under darkness in a solution consisting of a mixture of 4 mL phosphate buffer (pH = 7, 10 mM) and 40 μL of dihydrorhodamine (DHR123) previously dissolved in dimethyl sulfoxide (1 mM). Leaf samples were further submitted to vacuum infiltration inside a plastic syringe containing the same incubation medium for 15–20 s [40]. After infiltration, samples were washed twice in phosphate buffer (pH = 7, 10 mM) and then observed using a confocal laser-scanning microscope (Zeiss LSM 710 NLO, Germany). The DHR123 permits to quantify H_2_O_2_ since DHR123 interacts with H_2_O_2_ in leaf cells resulting in the formation of one rhodamine molecule (R123) and two molecules of H_2_O (DHR123 + H_2_O_2_ -> R123 + 2H_2_O) [41]. Once interaction occurred, rhodamine was excited using a 488 nm laser wavelength (λ) at 1% laser power, and maximum fluorescence re-emission of excited rhodamine was recorded at λ = 520 nm. Hence, a high fluorescence re-emission of excited rhodamine is an indicator of high H_2_O_2_ presence. Maximum autofluorescence of excited chlorophyll is reached at a wavelength of about λ = 685 nm. Previous tests using a laser excitation wavelength ranging from λ = 400 nm to λ = 700 nm allowed to check for maximal wavelengths where fluorescence showed maximum re-emission. Data collected were transformed into emitted fluorescence intensities from rhodamine and chlorophyll with total fluorescence corresponding to the sum of both rhodamine and chlorophyll fluorescence. Fluorescence spectra of rhodamine and chlorophyll were analyzed separately by spectral deconvolution. Fluorescence intensities of chlorophyll and rhodamine were obtained across the leaf thickness as the laser penetrated the leaf, which allowed to assess H_2_O_2_ location in upper (leaf cuticle and epidermis) and internal leaf tissues (mesophyll cells). Z-stack collections were carried out with optical section thickness of 2.5 µm, which also corresponds to the depth of each cut; thus, for example, cut 1 will be located in the cuticle and have 2.5 µm depth and cut 14 will be located deeper in the mesophyll cell and will have 35 µm depth. A modular image-processing and analysis software (ZEN 2) for digital microscopy was used for images analysis of rhodamine and chlorophyll fluorescence intensities. 

### 4.6. Statistical Analysis

A two-way analysis of variance followed by *post hoc* Tukey test tests were used to test for differences in the physiology of *A. monspessulanum* (*Pn*, *gs, Ci*) according to watering and fumigation treatments. When fumigation and water stress interacted significantly (*p* < 0.05, Table 2), a one-way ANOVA was applied to evaluate the physiological differences (i) between fumigations for each watering treatment and, accordingly, (ii) between watering treatments within each fumigation condition. Finally, variability of fluorescence intensities of rhodamine (indicator of cell oxidation) and chlorophyll according to the fumigation treatment was tested within each watering treatment using a Mann—Whitney test, and differences across watering treatments were tested within each fumigation treatment through Kruskal–Wallis tests. Statistical analyses were conducted with Statgraphics Centurion XVIII.

## 5. Conclusions

Previous investigations have provided evidence that isoprene-emitting plants are better protected against abiotic oxidative stress than negligible isoprene emitters (in practice considered as non-isoprene emitters). However, there is still a gap about the function that a high isoprene concentration in air, resulting from high isoprene emitters, has on negligible terpene emitters under drought conditions. Our study gives preliminary evidence that an isoprene-rich atmosphere confers protection to the model species *A. monspessulanum* by protecting significantly its photosynthetic apparatus under moderate water deficit through a reduction of the oxidative pressure all through the leaf depth. Thus, isoprene would be beneficial for isoprene emitters (as shown in literature) but also for neighboring non emitters (or target species) since isoprene emitted by one plant, once highly concentrated in the atmosphere, may provide protection after metabolization and/or deposition in the target species. Isoprene can thus be considered as a potential allelochemical with positive effects in physiology of target species that do not naturally produce it (or only in negligible amounts). Allelochemicals are indeed defined as compounds produced by organisms (including plants, micro-organisms, viruses and fungi), mostly through their secondary metabolism, that, once released into the environment, exert an influence (either negative or positive) on growth and development of other organisms (excluding mammals) ([42] and citations therein). Further research is necessary to evaluate *A. monspessulanum* responses to a range of isoprene concentrations under drought conditions. This approach would allow to assess whether this protective mechanism occurs over the entire phenological cycle when lower atmospheric isoprene concentrations are reached in the atmosphere of the forest canopy.

Since Mediterranean forest ecosystems feature high levels of ozone, temperature, and solar radiation as well as prolonged drought periods during summer months [43], we suggest that future works also tackle the influence of ambient isoprene on plant stress response when these abiotic constraints occur concomitantly. Isoprene, as the major atmospheric BVOC, could play a major functional role in these ecosystems by mitigating the negative effects of abiotic constraints in plant functioning. Plant species that emit terpenes in negligible amounts (such as *Acer* spp.), as members of complex communities, could thus have evolved intricate mechanisms involving protection conferred by BVOCs from the neighboring species under stressed climatic conditions.

## Figures and Tables

**Table 1:** Differences among the three watering treatments in terms of stem water potential (Ψ) and leaf water content tested with one-way ANOVA followed by post hoc Tukey test. ***: *p* < 0.001. Values shown correspond to the mean ± standard error (*n* = 4).

	Optimal Watering	Moderate Water Stress	Severe Water Stress	F-Value
Ψ (MPa)	−0.74a ± 0.18	−2.65b ± 0.21	−5.66c ± 0.39	78.63 ***
Leaf water content (%)	56.23a ± 0.98	20.56b ± 3.65	7.14c ± 1.33	120.15 ***

Different letters denote significant differences with a > b > c.

**Table 2:** Effect of watering and fumigation on net photosynthesis (*Pn*), stomatal conductance to water (gs*)* and intercellular CO_2_ concentration (*Ci*). Values shown correspond to the ANOVA value. *: 0.01 < *p* < 0.05, ***: *p* < 0.001. ns: not significant. *n* = 5.

	*Pn*	*gs*	*Ci*
Fumigation	3.2 *	0.01ns	0.3ns
Watering	143.7 ***	47.8 ***	386.3 ***
Watering x Fumigation	4.9 *	0.16ns	0.5ns
